# *Scutellaria baicalensis* Georgi regulates REV-ERBα/BMAL1 to protect against skin aging in mice

**DOI:** 10.3389/fphar.2022.991917

**Published:** 2022-09-30

**Authors:** Guanghui Sun, Yongkang Dang, Yanke Lin, Wanying Zeng, Zongjian Wu, Xingwang Zhang, Dong Dong, Baojian Wu

**Affiliations:** ^1^ College of Pharmacy, Jinan University, Guangzhou, China; ^2^ Institute of Molecular Rhythm and Metabolism, Guangzhou University of Chinese Medicine, Guangzhou, China; ^3^ School of Medicine, Jinan University, Guangzhou, China

**Keywords:** scutellaria baicalensis georgi, skin aging, REV-erbα, BMAL1, photoaging

## Abstract

*Scutellaria baicalensis* Georgi (SBG) is a traditional Chinese medicine widely used to treat disorders such as hypertension, dysentery and hemorrhaging. Here, we aimed to assess the pharmacological effects of SBG on skin aging and to investigate the underlying mechanisms. Mice with skin aging were established by treatment with D-galactose and ultraviolet-B. SBG (topical application) showed a protective effect on skin aging in mice, as evidenced by less formation of skin wrinkles, higher levels of SOD (superoxide dismutase) and HYP (hydroxyproline) as well as a lower level of MDA (malondialdehyde). In the meantime, skin MMP-1 and p53 expression were lower, epidermis was thinner and collagen amount was higher in SBG-treated mice. Anti-skin aging effects of SBG were also confirmed in NIH3T3 and HaCaT cells, as well as in mouse primary dermal fibroblasts and human primary epidermal keratinocytes. Furthermore, we found that loss of *Rev-erbα* (a known repressor of Bmal1) up-regulated skin BMAL1 (a clock component and a known anti-aging factor) and ameliorated skin aging in mice. Moreover, SBG dose-dependently increased the expression of BMAL1 in the skin of aged mice and in senescent NIT3H3 cells. In addition, based on a combination of Gal4 chimeric, luciferase reporter and expression assays, SBG was identified as an antagonist of REV-ERBα and thus an inducer of BMAL1 expression. In conclusion, SBG antagonizes REV-ERBα to up-regulate BMAL1 and to protect against skin aging in mice.

## Introduction

*Scutellaria baicalensis* Georgi (SBG, also known as *Huangqin* in Chinese), a traditional Chinese medicine, possesses various pharmacological effects such as anti-inflammatory, antiviral, anticancer, anti-oxidant and antibacterial activities. SBG is widely used to treat diarrhea, hypertension, dysentery and hemorrhaging (Liao et al., 2021). Many types of chemicals are found in SBG, including flavones, phenylethanoids, amino acids, sterols and essential oils (Zhao et al., 2016). Of note, flavones (e.g., baicalin, baicalein, wogonin and oroxylin A) are thought to be a major class of active ingredients of SBG as they show the health-promoting effects (such as anti-inflammation, antivirus, anticancer, anti-oxidation and anti-bacteria) typically observed for SBG (Brown, 1980; Li et al., 2004). It is interesting to note that SBG has great potential to manage the skin diseases caused by sunlight irradiation (Brown, 1980). The underlying mechanisms may involve scavenging of free radicals and attenuation of lipid oxidation (Gabrielska et al., 1997). However, it remains unknown whether SBG can protect against skin aging.

Skin aging is classified into intrinsic (chronological) and extrinsic aging, and the latter is also referred to as premature skin aging or photoaging (Bocheva et al., 2019). Intrinsic aging is an unpreventable spontaneous process, whereas extrinsic aging caused by exogenous factors (e.g., ultraviolet/UV light, cigarette smoking and pollution) is preventable (Fisher et al., 2002; Bernhard et al., 2007). UV radiation is a major cause of photoaging, and can be divided into three bands [i.e., UVA (315–400 nm), UVB (280–315 nm) and UVC (100–280 nm)]. Of note, UVB is the predominant form that causes injuries to living organisms (Slominski et al., 2018a; Frommeyer et al., 2022). UV radiation not only induces skin pathology, but also exert systemic effects, including activation of hypothalamic-pituitary-adrenal axis, opioidogenic effects, and immunosuppression. Thus, UV radiation has therapeutic applications in management of various diseases such as addiction, autoimmune and mood disorders (Slominski et al., 2018a). Although skin aging is regarded as a cosmetic problem, it can result in disfigurement and skin diseases (such as skin cancers) and has profound psychological consequences (Watson et al., 2016; Narayanan et al., 2010). There are two major classes of agents for management of skin aging, namely, antioxidants and cell regulators. However, these medications (e.g., retinoid) are concerned with the lack of effectiveness and/or adverse effects (Krutmann et al., 2021; Mukherjee et al., 2006). Therefore, it is of value to search for more effective and safer therapeutic agents.

BMAL1 (Brain and muscle ARNT-like protein 1) is a transcription factor and a core component of circadian clock system, which generates and maintains circadian rhythms in most aspects of physiology and behaviors (Gekakis et al., 1998; Hogenesch et al., 1998; Bunger et al., 2000). *Bmal1* and other clock genes work cooperatively to drive circadian gene expression using a negative feedback mechanism (Chen et al., 2009; Duong et al., 2011). BMAL1 forms a heterodimer with CLOCK (circadian locomotor output cycles kaput) to activate the transcription of *Pers* (periods) and *Crys* (cryptochromes) as well as many other clock-controlled genes (CCGs) (Langmesser et al., 2008). Once reaching a critical level, PER and CRY proteins in turn inhibit the activity of the BMAL1/CLOCK dimer, bringing down the levels of CCGs (Böger, 2014). As PER and CRY proteins are reduced due to degradation, a new cycle of BMAL1/CLOCK-driven transcription can begin (Duong et al., 2011). In addition to regulating circadian rhythms, BMAL1 plays a role in the development and progression of many types of diseases such as cancers (Jung et al., 2013), obesity (Hemmeryckx et al., 2011), and neurodegenerative disorders (Vieira et al., 2020). Notably, *Bmal1* is also involved in aging (Khapre et al., 2011). *Bmal1*-deficient mice have reduced lifespan and are prone to premature aging (exemplified by sarcopenia, cataracts, reduced subcutaneous fat, decreased organ size and impaired hair growth) (Kondratov et al., 2006). *Bmal1* regulates aging *via* modulation of the expression of major antioxidant enzymes including SOD, peroxiredoxines and glutathione peroxidase (Kondratov et al., 2009).

REV-ERBα (also known as NR1D1, nuclear receptor subfamily one group D member 1) is a nuclear receptor that participates in regulation of circadian rhythms *via* inhibiting BMAL1 expression (Yin and Lazar, 2005). REV-ERBα functions as a transcriptional repressor that inhibits the transcription of target genes (e.g., *Bmal1*) by binding to a response element (called RevRE) in the promoters and recruiting the corepressors nuclear corepressor one and histone deacetylase 3 (Liu et al., 2008; Yin et al., 2010). REV-ERBα has been also implicated in regulation of a variety of diseases including inflammatory diseases (e.g., fulminant hepatitis, pulmonary inflammation, and colitis) (Wang et al., 2020), metabolic disorders (Delezie et al., 2012) and cancers (Wang et al., 2015). SR8278 (a synthetic compound) is identified as an antagonist of REV-ERBα and widely used to probe the function of REV-ERBα (Kojetin et al., 2011; Pardee et al., 2011). Notably, we recently found that REV-ERBα restrains *Propionibacterium acnes*-induced skin inflammation through inhibiting the NF-κB/NLRP3 axis to protect against acne vulgaris (Li et al., 2022). However, it remains elusive whether and how REV-ERBα regulates skin aging.

In the present study, we aimed to assess the pharmacological effects of SBG on skin aging and to investigate the underlying mechanisms. Anti-skin aging effects of SBG were evaluated using mouse and cell models of aging (induced by D-galactose and/or ultraviolet-B). Skin aging was assessed by analyzing SOD, HYP, MDA, ROS and MMP-1/p53 and by measuring epidermal thickness and collagen content. The role of REV-ERBα/BMAL1 in regulating skin aging was assessed using gene knockout mice. Antagonism of REV-ERBα was determined using Gal4 chimeric assay. We for the first time demonstrated that SBG antagonizes REV-ERBα to up-regulate BMAL1 (a skin aging-inhibiting factor) and to protect against skin aging in mice.

## Materials and methods

### Materials

SBG was purchased from Biopurify Phytochemicals (Chengdu, China). D-galactose (D-gal) was purchased from Aladdin (Shanghai, China). Vitamin C was obtained from Yuanye Biotechnology (Shanghai, China). Biochemical kits for SOD, MDA, ROS and HYP were purchased from Jiancheng Bioengineering Institute (Nanjing, Jiangsu, China). Staining kit for senescence-associated-β-galactosidase (SA-β-gal) was obtained from Beyotime Biotechnology (Shanghai, China). Antibodies against GAPDH, MMP-1, p53, BMAL1, REV-ERBɑ, BHMT and NLRP3 were purchased from Abcam (Cambridge, United Kingdom).

### Preparation of SBG extract

SBG was extracted for 90 min by refluxing in 80% ethanol (3:20, *w*/*v*), and filtered with filter paper. The filtrate was concentrated and freeze-dried. The dry residue (SBG extract) was stored at -20°C. The extraction yield was 23.3%, and the main active ingredients of SBG are shown in Supplementary Table S1. For animal experiments, SBG extract was mixed with a homemade cream containing stearic acid, triethanolamine, and propylene glycol.

### Animals

C57BL/6 mice (10 weeks old) weighing 18–22 g were obtained from HFK Bioscience (Beijing, China). *Rev-erbɑ* ^*−/−*^ mice (on a C57BL/6 background) have been established and validated in our laboratory (Wang et al., 2018). All mice were maintained on a 12 h light/12 h dark cycle, with free access to food and water. Mice were individually placed in the cages to prevent offensive behaviors from other mice, that may cause injuries to the skin. Mice from the same litter (with hair growth in the anagen phase) were used for experiments. Note that we used male mice to assess the therapeutic effect of SBG on skin aging, without considering factors such as hormone-induced wrinkling of skin. Protocols for animal experiments were approved by the Institutional Animal Care and Use Committee of Guangzhou University of Chinese Medicine (Appr. Date: 2021–05-17; IACUC Issue No: ZYD-2021–112).

### LC-MS/MS analysis

The main active constituents (i.e., wogonoside, baicalein, baicalin and wogonin) in SBG extract were quantified using a Shimadzu LCMS-8045 triple quadrupole liquid chromatograph mass spectrometer (LC-MS) equipped with Shimadzu-Nexera XR high-performance liquid chromatography (HPLC). The mobile phases consisted of acetonitrile (A) and water (B). Flow rate was set at 0.3 ml/min. Gradient elution program was 40% B (0–1 min), 40–10% B (1–3 min), 10% B (3–4 min) and 10–40% B (4–5 min). Mass spectrometer was operated at positive ion scan mode. The mass transition ion pairs and contents of main active constituents are provided in Supplementary Table S1

### Mouse model of skin aging and drug treatment

To induce skin aging, mice were injected subcutaneously with 250 mg/kg D-gal daily in the back neck and irradiated on the back daily with 120 mJ/cm^2^ UVB for 6 weeks as previously described (Zhang et al., 2020). The source of radiation was a narrow band UVB bulb (Philips model PL-9 9W/01/2P) emitting photons with wavelengths between 306 and 316 nm, with a peak at 312 nm. The distance from the UVB lamp (KN-4003BL, Kernel Medical Equipment, Xuzhou, China) to the mouse back was 25 cm. Control mice were injected subcutaneously with vehicle (saline). To assess the effects of SBG on skin aging, SBG extract (25, 100 or 400 mg/kg), vitamin C (40 mg/kg) or vehicle was applied topically (once daily after UVB exposure) on the skin of mouse models for 4 weeks from the third week. Mice were sacrificed to collect skin samples, followed by qPCR, Western blotting and biochemical analyses (SOD, MDA and HYP).

### Isolation of mouse primary dermal fibroblasts

Mouse primary dermal fibroblasts were isolated from newborn mice as previously described (Terao et al., 2014). The newborn mice were sacrificed by rapid cervical dislocation. Trunk skin was peeled off and incubated with 4 mg/ml dispase overnight at 4 °C. On the next day, the dermis was separated from the epidermis using forceps, and incubated with 0.25% trypsin for 10 min. After filtration, cells were centrifuged at 200 g for 10 min, resuspended in Dulbecco’s modified Eagle’s medium (DMEM) supplemented with 10% fetal bovine serum (FBS) and incubated at 37°C and 5% CO_2_.

### Cell culture and treatment

NIH3T3, HaCaT and Primary adult human epidermal keratinocytes (HEKa) cells were obtained from the American Type Culture Collection (Rockville, MD). NIH3T3, HaCaT and HEKa were maintained in DMEM supplemented with 10% FBS, 100 U/ml penicillin and 100 mg/ml streptomycin. To induce NIH3T3 cell senescence, 8 g/L D-gal was added to the culture medium for 96 h. To induce cell senescence of HaCaT, mouse primary dermal fibroblasts and HEKa, cells were subjected to UVB irradiation (100 mJ/cm^2^) with a thin layer of PBS (phosphate-buffered saline) using a UVB lamp (KN-4003BL, Kernel Medical Equipment, Xuzhou, China). Cells were then treated with SBG or vehicle. On next day, cells were collected for qPCR and Western blotting.

### Gal4 co-transfection assay

Gal4 co-transfectionthe assay was performed as previously described (Zhang et al., 2018). In brief, HEK293 cells were co-transfected with pGal4-Rev-erbα-LBD plasmid (200 ng), pGL-4.35-Luc reporter (100 ng, a Gal4-reponsive luciferase reporter) and pRL-TK vector (10 ng) using jetPRIME (Polyplus Transfection, Illkirch, France). On next day, cells were treated with SBG or SR8278 or vehicle. 24 h later, luciferase activities were measured using the Dual-Luciferase Reporter Assay system and GloMax 20/20 luminometer (Promega Madison, WI).

### Luciferase reporter assays

Luciferase reporter assays were performed as previously described (Chen et al., 2020). In brief, NIH3T3 cells were cultured in DMEM medium (containing 10% FBS, 1% penicillin-streptomycin) and transfected with 250 ng of Bmal1 luciferase reporter plasmid and 50 ng of pRL-TK using jetPRIME (Polyplus Transfection, Illkirch, France). On next day, SBG or SR8278 or vehicle was added to the culture medium for 24 h. Cells were harvested and lysed with passive lysis buffer. Luciferase activities were detected using the Dual-Luciferase Reporter Assay system and GloMax 20/20 luminometer (Promega, Madison, WI).

### H&E and masson staining

Skin samples were fixed in 10% formalin, embedded in paraffin, and cut to 4 μm-thick sections. The sections were subjected to H&E (hematoxylin and eosin) and Masson staining. The images were captured using an optical microscope (Olympus, Tokyo, Japan).

### Measurement of intracellular ROS

Cells were treated with 2′7′-dichlorodihydrofluorescein diacetate (10 μM) at 37°C for 1 h. Fluorescence intensity was determined at excitation (485 nm) and emission (530 nm) wavelengths using a Synergy HT Multi-Mode Microplate Reader (BioTek, Winooski, VT).

### MTT assay

MTT assays were performed to determine cell viability as previously described (Kumar et al., 2018). Briefly, cells were incubated with MTT [3-(4,5-dimethylthiazol-2-yl)-2,5 diphenyl tetrazolium bromide, 0.5 mg/ml] for 4 h, and the formazan crystals were dissolved in 100 μl DMSO. The absorbance was determined at 570 nm using a Synergy HT Multi-Mode Microplate Reader (BioTek, Winooski, VT).

### SA-β-gal assay

SA-β-gal activity was measured using a SA-β-gal staining kit according to the manufacturer’s instructions (Beyotime, Shanghai, China). Briefly, cells were fixed in a fixing solution for 15 min, washed with PBS and incubated with senescence detection solution at 37°C. On next day, the number of SA-β-gal-positive cells were determined by counting blue-stained cells using an Eclipse ci-L microscope (Nikon, Tokyo, Japan).

### Real-time luminescence monitoring

Real-time luminescence monitoring was performed as previously described (Hirota et al., 2008; du Pré et al., 2017). In brief, NIH3T3 cells stably overexpressed with *Bmal1*-*dLuc* were seeded into a 35 mm dish and maintained in DMEM containing 10% FBS. On next day, cells were incubated with a recording medium containing 1 μg/ml SBG or vehicle. Luminescence data (counts/s) were collected by using Lumicycle 32 (Actimetrics, Wilmette, IL).

### Western blotting

Protein samples were subjected to 10% sodium dodecyl sulfate-polyacrylamide gel electrophoresis (SDS-PAGE), and transferred to PVDF membranes. The membranes were blocked with 5% skim milk, and sequentially incubated with primary and secondary antibodies. Protein bands were visualized by using enhanced chemiluminescence and Omega Lum G imaging system (Aplegen, Pleasanton, CA), and quantified with Fluorchem 5500 software (Fisher Scientific, Fair Lawn, NJ). GAPDH was used as a loading control.

### qPCR (quantitative polymerase chain reaction)

RNA was extracted using RNAiso Plus reagent (Takara, Shiga, Japan) and transcribed to cDNA using PrimeScript RT Master Mix (Vazyme, Jiangsu, China). qPCR reactions were performed using the SYBR Premix Ex Taq (Vazyme, Jiangsu, China). Amplification procedures consisted of an initial denaturation at 95°C for 5 min, 40 cycles of denaturation at 95°C for 15 s, annealing at 60°C for 30 s, and extension at 72°C for 30 s. Mouse *Gapdh* was used as an internal control. Gene expression was determined using the 2^−ΔΔCT^ method. Primers are provided in Table 1.

**TABLE 1:** Primers used for qPCR assays.

Gene	Forward (5–3′)	Reverse (5–3′)
*mp53*	GTC​ACA​GCA​CAT​GAC​GGA​GG	TCT​TCC​AGA​TGC​TCG​GGA​TAC
*mMmp-1*	CTT​CTT​CTT​GTT​GAG​CTG​GAC​TC	CTG​TGG​AGG​TCA​CTG​TAG​ACT
*mBhmt*	TTA​GAA​CGC​TTA​AAT​GCC​GGA​G	GAT​GAA​GCT​GAC​GAA​CTG​CCT
*mNlrp3*	ATT​ACC​CGC​CCG​AGA​AAG​G	TCG​CAG​CAA​AGA​TCC​ACA​CAG
*mRev-erbɑ*	TTT​TTC​GCC​GGA​GCA​TCC​AA	ATC​TCG​GCA​AGC​ATC​CGT​TG
*mBmal1*	CTC​CAG​GAG​GCA​AGA​AGA​TTC	ATA​GTC​CAG​TGG​AAG​GAA​TG
*mGapdh*	CAA​GGA​GTA​AGA​AAC​CCT​GGA	CGA​GTT​GGG​ATA​GGG​CCT​CT
*hp53*	TTG​GCT​CTG​ACT​GTA​CCA​CCA​T	CAG​TGT​GAT​GAT​GGT​GAG​GAT​G
*hMMP-1*	TCG​GGG​CTT​TGA​TGT​ACC​CT	ACA​CGC​TTT​TGG​GGT​TTG​TG
*hGAPDH*	CAT​GAG​AAG​TAT​GAC​AAC​AGC​CT	TAG​TCC​TTC​CAC​GAT​ACC​AAA​GT

m, mouse; h, human.

### Statistical analysis

Data are presented as means ± SD (standard deviation). Comparisons between two groups were analyzed using Student’s t-test. One-way ANOVA followed by Bonferroni post hoc test was performed to compare means of more than two groups. The level of significance was set at *p* < 0.05 (*).

## Results

### SBG protects against skin aging induced by D-gal/UVB in mice

**FIGURE 1:**
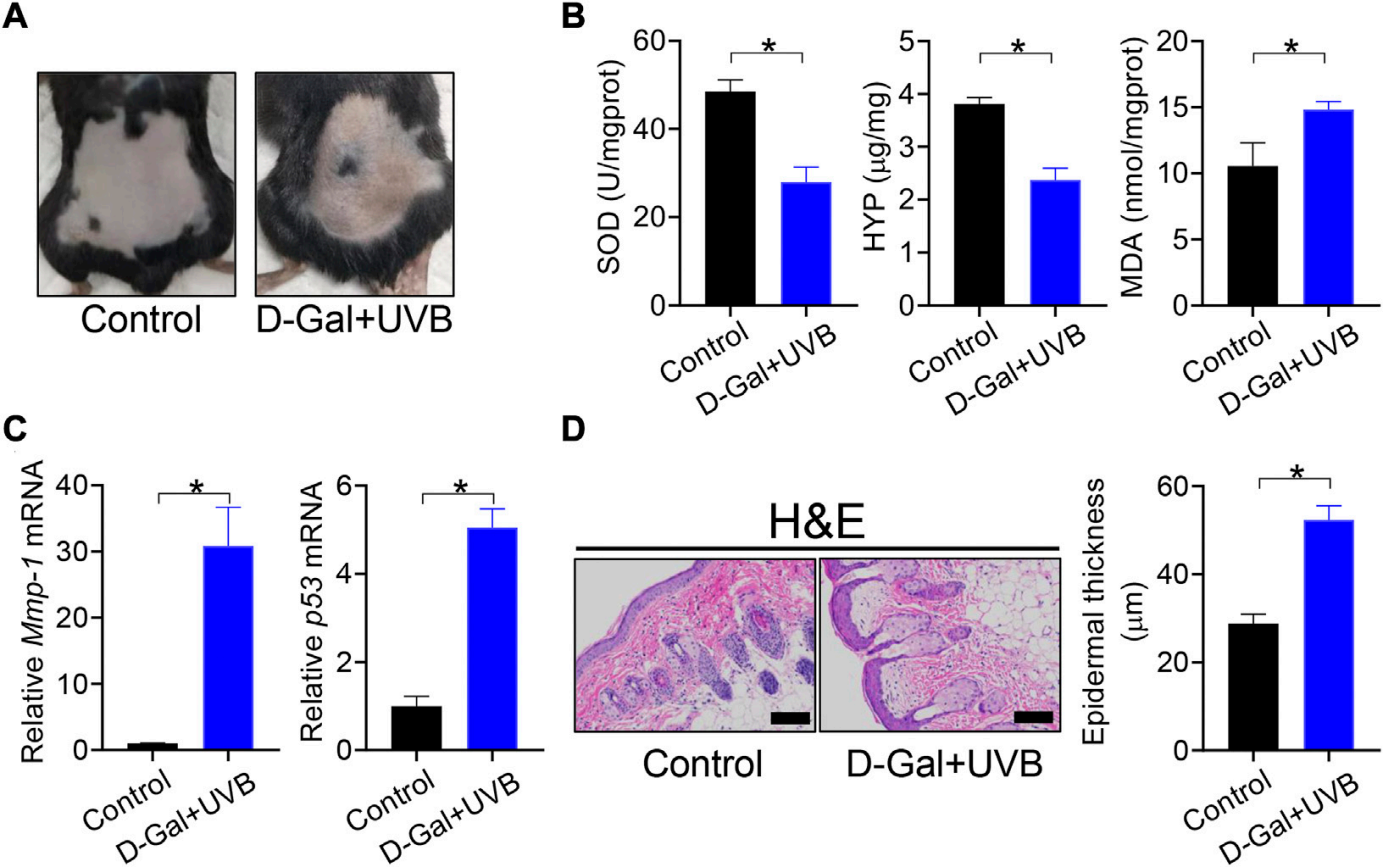
Establishment of mice with skin aging**. (A)** Surface examination of dorsal skin derived from D-gal/UVB-treated and control mice. **(B)** Skin SOD activity and HYP/MDA levels in D-gal/UVB-treated and control mice. Data are mean ± SD (*n* = 7). *p < 0.05 (*t*-test). **(C)** mRNA expression of *Mmp-1* and *p53* in the skin of D-gal/UVB-treated and control mice. Data are mean ± SD (*n* = 7). *p < 0.05 (*t*-test). **(D)** H&E staining (left panel) and epidermal thickness (right panel) of skin derived from D-gal/UVB-treated and control mice. Scale bar = 100 μm.

Mice with skin aging were established by treatment with D-gal/UVB as previously described (du Pré et al., 2017). As expected, D-gal/UVB-treated mice showed skin aging-like symptoms such as roughness, stiffness, and lack of elasticity (Figure 1A). These mice had lower levels of SOD activity and HYP and a higher level of MDA in the skin as compared to normal mice (Figure 1B). We also observed marked increases in the expression of *Mmp-1* and *p53* (two genes closely associated with skin aging) in the skin of D-gal/UVB-treated mice (Figure 1C). In addition, the epidermis (D-gal/UVB-exposed region) was thicker in aging mice than in control mice according to H&E staining (Figure 1D). These data indicated successful construction of mice with skin aging.

**FIGURE 2:**
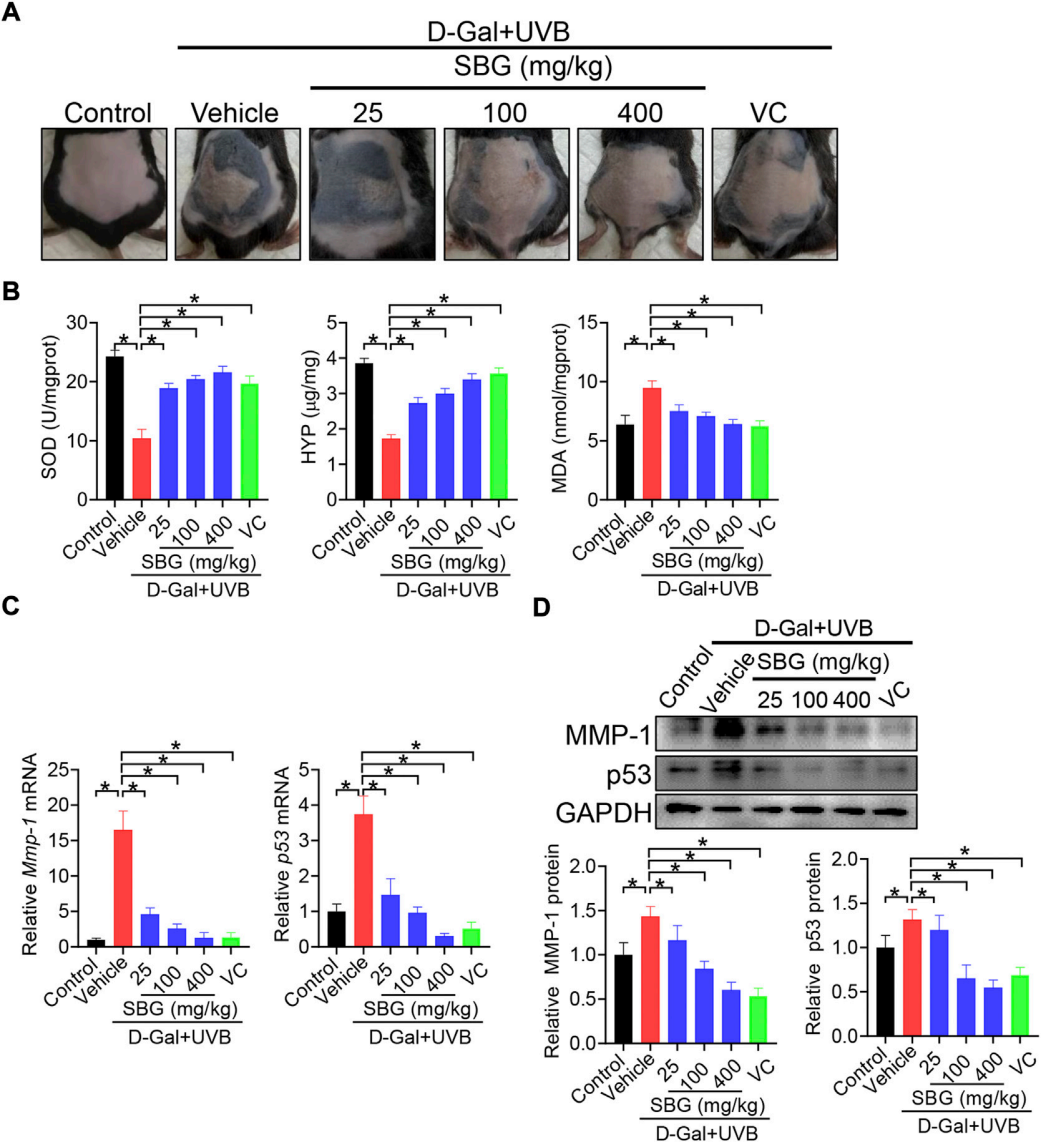
SBG protects against skin aging induced by D-gal/UVB in mice. **(A)** Surface examination of dorsal skin derived from D-gal/UVB-treated and control mice. **(B)** Skin SOD activity and HYP/MDA levels in D-gal/UVB-treated and control mice. **(C)** mRNA expression of *Mmp-1* and *p53* in the skin of D-gal/UVB-treated and control mice. **(D)** Protein expression (top panel) and quantification (bottom panel) of MMP-1/p53 in the skin of D-gal/UVB-treated and control mice. In panels B–D, data are mean ± SD (*n* = 7). *p < 0.05 (one-way ANOVA and Bonferroni post hoc test). VC, vitamin.

**FIGURE 3:**
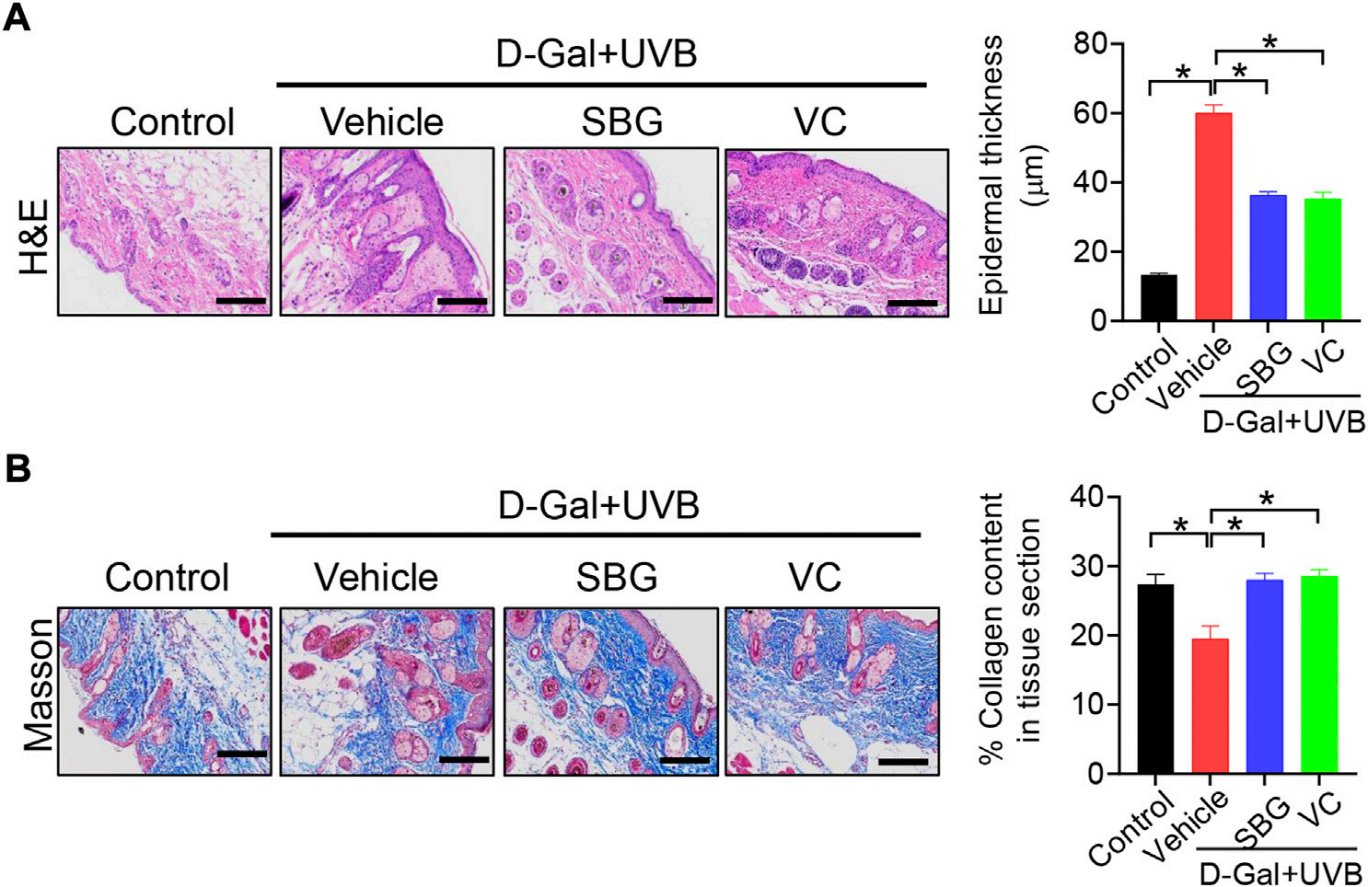
Effects of SBG on epidermal thickness and collagen content in aging mice. **(A)** H&E staining (left panel) and epidermal thickness (right panel) of skin derived from D-gal/UVB-treated and control mice. Scale bar = 100 μm. **(B)** Masson staining of the skin derived from D-gal/UVB-treated and control mice. The right panel shows collagen content in the skin. Data are mean ± SD (*n* = 7). *p < 0.05 (one-way ANOVA and Bonferroni post hoc test). Scale bar = 100 μm.

We next assessed the effects of SBG (topical application) on skin aging in mice. Like vitamin C (a known anti-aging agent and used as a positive control), SBG reduced the wrinkle formation in the skin of D-gal/UVB-treated mice (Figure 2A). Further, SBG dose-dependently increased the SOD activity and HYP level, and decreased the MDA level in the skin of aging mice (Figure 2B). Moreover, SBG reduced the mRNAs and proteins of both MMP-1 and p53 in the skin of aging mice in a dose-dependent manner (Figure 2C/D). In addition, compared to vehicle-treated aging mice, SBG-treated mice had thinner D-gal/UVB-exposed epidermis, and a higher amount of collagen (Figure 3A/B). Taken together, these findings clearly indicated a protective effect of SBG on skin aging in mice.

### SBG attenuates D-gal- and UVB-induced cell senescence

**FIGURE 4:**
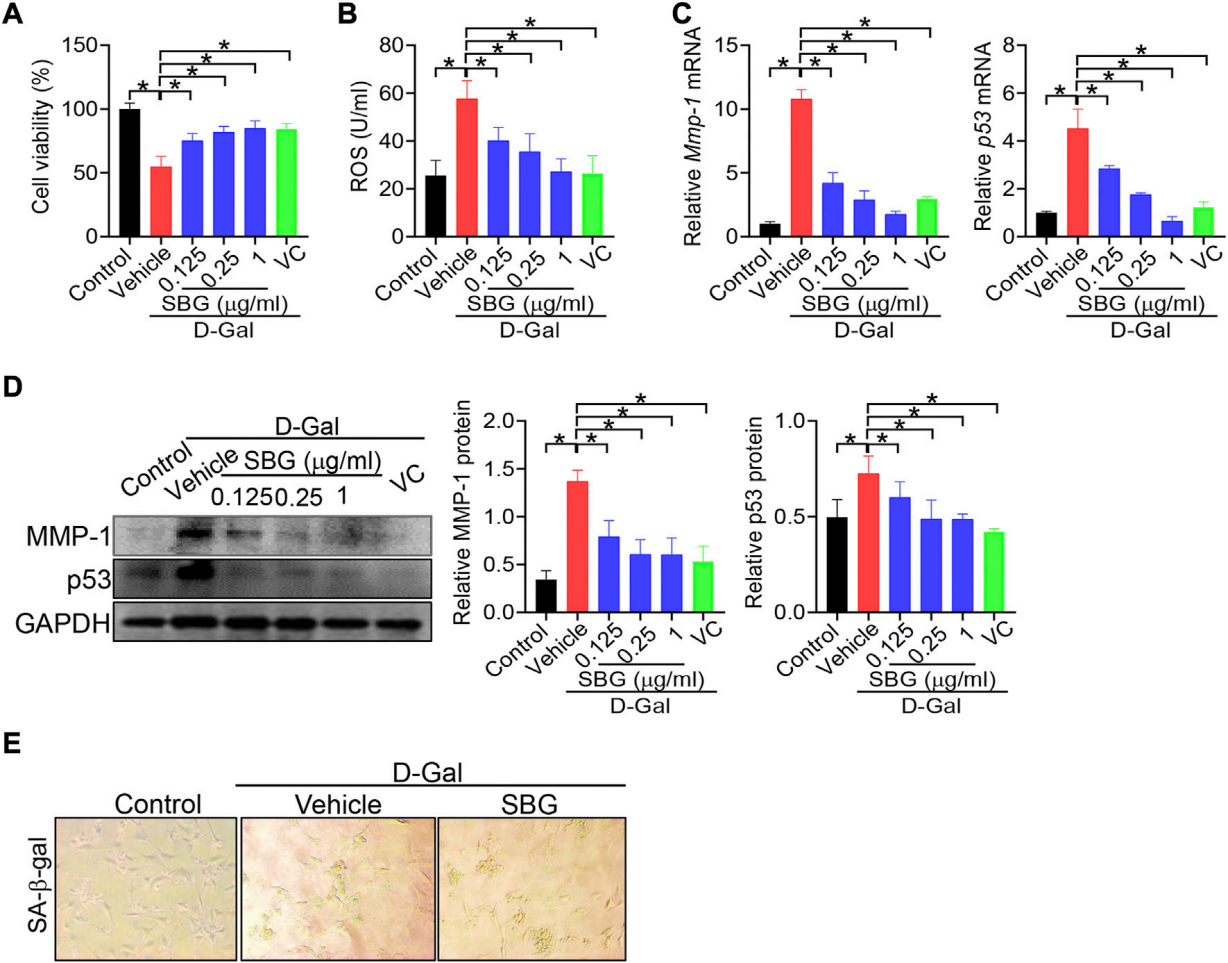
SBG attenuates D-gal-induced senescence in NIH3T3 cells**. (A)** Effects of SBG on the viability of D-gal-treated NIH3T3 cells. **(B)** Effects of SBG on ROS accumulation in D-gal-treated NIH3T3 cells. **(C)** Effects of SBG on *Mmp-1* (left panel) and *p53* (right panel) mRNA expression in D-gal-treated NIH3T3 cells. **(D)** Effects of SBG on protein expression of MMP-1 and p53 in D-gal-treated NIH3T3 cells. **(E)** Representative images showing SA-β-Gal activity in SBG-treated senescent NIH3T3 cells. In panels A–D, data are mean ± SD (*n* = 3). *p < 0.05 (one-way ANOVA and Bonferroni post hoc test).

We next investigated whether SBG can affect cell senescence using NIH3T3 and HaCaT cells. NIH3T3 cells were exposed to 8 g/L D-gal for 96 h to induce cell senescence as previously described (Gao et al., 2014). As expected, D-gal treatment of NIH3T3 cells resulted in senescence as evidenced by a decrease in cell viability, and elevations in ROS level and MMP-1/p53 expression, as well as an increase in senescence-associated β-galactosidase (SA-β-gal, a senescence-specific marker) activity (Figure 4). We found that SBG dose-dependently increased the cell viability and decreased the ROS level in D-gal-treated NIH3T3 cells (Figure 4A/B). Furthermore, SBG down-regulated the mRNA levels of both *Mmp-1* and *p53* in a dose-dependent fashion in D-gal-treated cells (Figure 4C). In line with the mRNA changes, MMP-1 and p53 proteins were reduced by SBG in the aged cells (Figure 4D). Moreover, SBG-treated senescent cells had a lower activity of SA-β-gal (Figure 4E).

**FIGURE 5:**
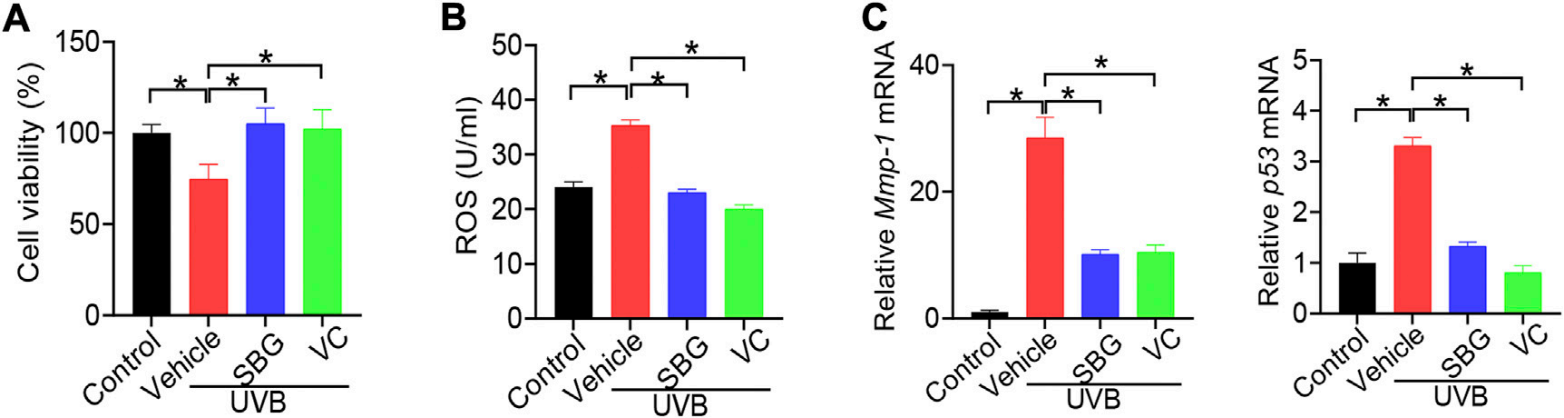
SBG attenuates UVB-induced senescence in HaCaT cells**. (A)** Effects of SBG on the viability of UVB-treated HaCaT cells. **(B)** Effects of SBG on ROS accumulation in UVB-treated HaCaT cells**. (C)** Effects of SBG on the mRNA expression of *Mmp-1* and *p53* in UVB-treated HeCaT cells. Data are mean ± SD (*n* = 3). *p < 0.05 (one-way ANOVA and Bonferroni post hoc test).

**FIGURE 6:**
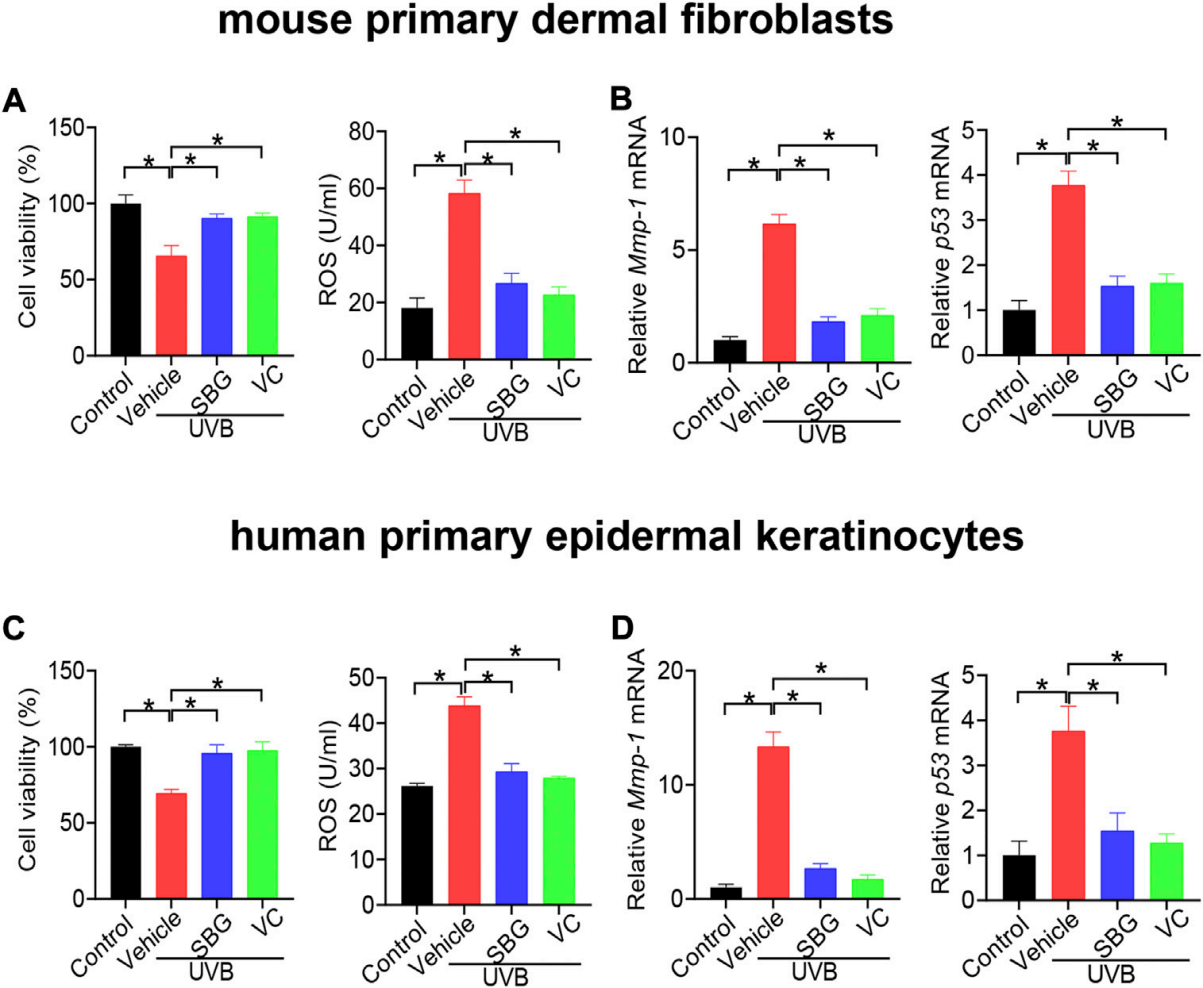
SBG attenuates UVB-induced senescence in mouse primary dermal fibroblasts and HEKa cells**. (A)** Effects of SBG on cell viability and ROS accumulation for UVB-treated mouse primary dermal fibroblasts. **(B)** Effects of SBG on mRNA expression of *Mmp-1* and *p53* in UVB-treated mouse primary dermal fibroblasts. **(C)** Effects of SBG on cell viability and ROS accumulation for UVB-treated HEKa cells. **(D)** Effects of SBG on mRNA expression of *Mmp-1* and *p53* in UVB-treated HEKa cells. Data are mean ± SD (*n* = 3). *p < 0.05 (one-way ANOVA and Bonferroni post hoc test).

We additionally examined the effects of SBG on senescene of HaCaT, mouse primary dermal fibroblasts and HEKa cells induced by UVB. SBG increased the cell viability and decreased the ROS level and MMP-1/p53 expression in the senescent cells (Figures 5, 6). These similar effects were also observed for vitamin C (Figures 5, 6). Altogether, these findings supported that SBG had a protective effect on skin aging.

### Loss of *Rev-erbα* up-regulates skin BMAL1 and ameliorates skin aging in mice

**FIGURE 7:**
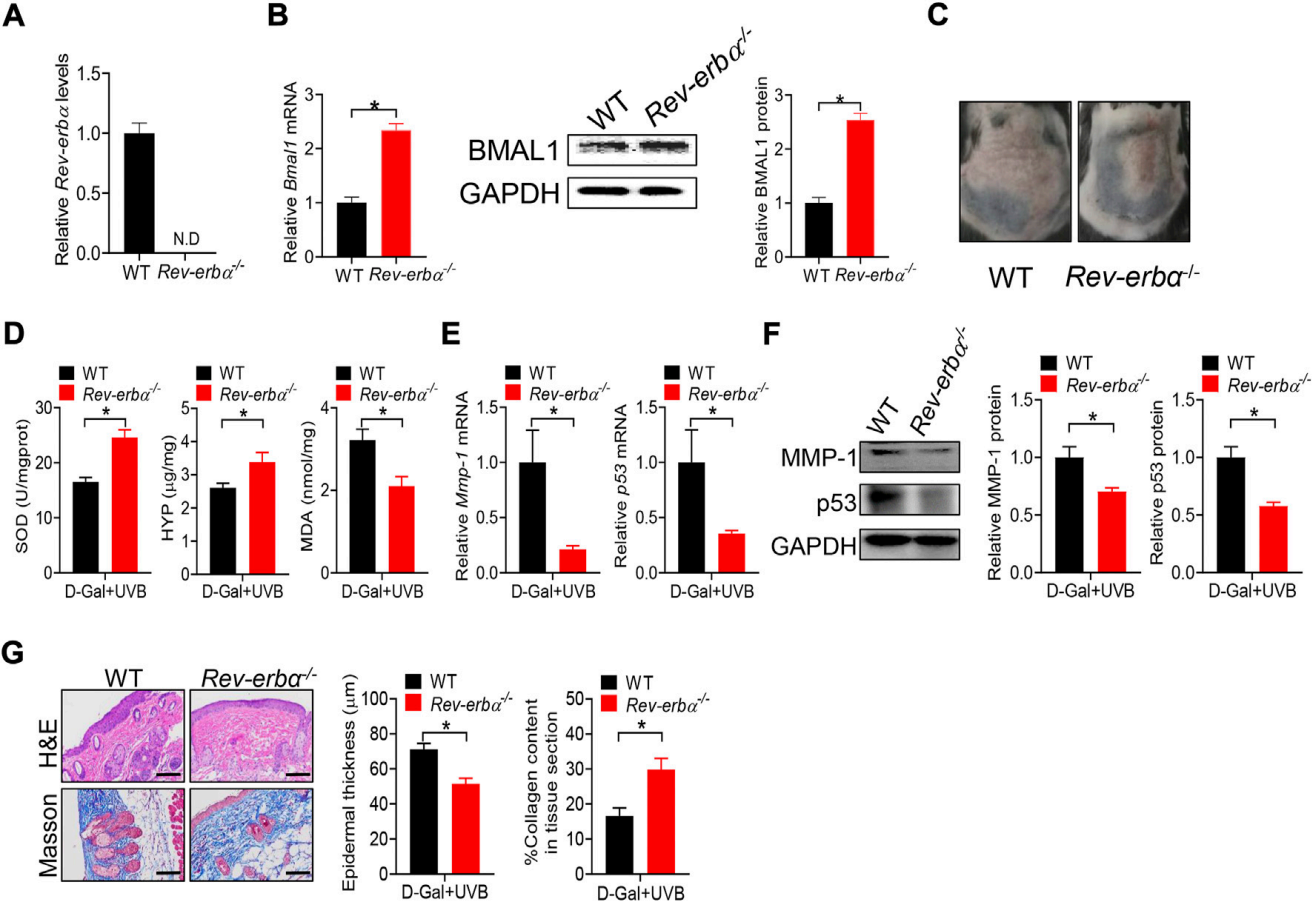
Loss of *Rev-erbα* up-regulates skin BMAL1 and ameliorates skin aging in mice**. (A)** mRNA expression of *Rev-erbα* in skin derived from *Rev-erbα* ^*−/−*^ and wild-type (WT) mice. **(B)** mRNA (left panel) and protein (right panel) expression of BMAL1 in skin derived from *Rev-erbα* ^*−/−*^ and WT mice. **(C)** Surface examination of dorsal skin derived from *Rev-erbα* ^*−/−*^ and WT mice treated with D-gal/UVB. **(D)** Skin SOD activity and HYP/MDA levels in skin derived from *Rev-erbα* ^*−/−*^ and WT mice treated with D-gal/UVB. **(E)** mRNA expression of *Mmp-1* and *p53* in the skin derived from *Rev-erbα* ^*−/−*^ and WT mice treated with D-gal/UVB. **(F)** Protein expression of MMP-1/p53 in skin derived from *Rev-erbα* ^*−/−*^ and WT mice treated with D-gal/UVB. **(G)** H&E and Masson staining showing epidermal thickness and collagen content in skin derived from *Rev-erbα* ^*−/−*^ and WT mice treated with D-gal/UVB. In panels A–B, data are mean ± SD (*n* = 3). In panels C–G, data are mean ± SD (*n* = 7). *p < 0.05 (*t*-test).

Deficiency of *Bmal1* has been previously shown to promote premature aging in mice, identifying *Bmal1* as a key regulator of aging (Kondratov et al., 2006). *Bmal1* regulates aging *via* modulation of the expression of antioxidant enzymes including SOD, peroxiredoxines and glutathione peroxidase (Kondratov et al., 2009). *Bmal1* is a target gene of REV-ERBα, which functions as a transcriptional repressor (Crumbley and Burris, 2011). Loss of *Rev-erbα* leads to up-regulation of BMAL1 (Burris, 2008). Thus, we hypothesized that *Rev-erbα* may promote skin aging considering that *Bmal1* has a protective role. To test this hypothesis, we generated and tested a mouse line with deletion of *Rev-erbα* gene (*Rev-erbα* ^*−/−*^ mice). *Rev-erbα* ^*−/−*^ mice were validated by qPCR, and showed the absence of wild-type *Rev-erbα* transcript in the skin (Figure 7A)*.* As expected, BMAL1 mRNA and protein were up-regulated in the skin of *Rev-erbα* ^*−/−*^ mice (Figure 7B). *Rev-erbα* ^*−/−*^ and control mice were subjected to induction of skin aging by D-gal/UVB. We found that loss of *Rev-erbα* ameliorated skin aging as evidenced by less formation of skin wrinkles, higher levels of SOD and HYP as well as a lower level of MDA (Figure 7C/D). In the meantime, skin MMP-1 and p53 expression were lower, epidermis was thinner and collagen amount was higher in *Rev-erbα* ^*−/−*^ mice (Figures 7E–G)*.* Taken together, *Rev-erbα* ablation up-regulated skin BMAL1 and ameliorated skin aging in mice. Our findings supported a critical role of REV-ERBα/BMAL1 in regulation of skin aging.

### SBG up-regulates the clock gene *Bmal1* in mouse and cell models of aging

**FIGURE 8:**
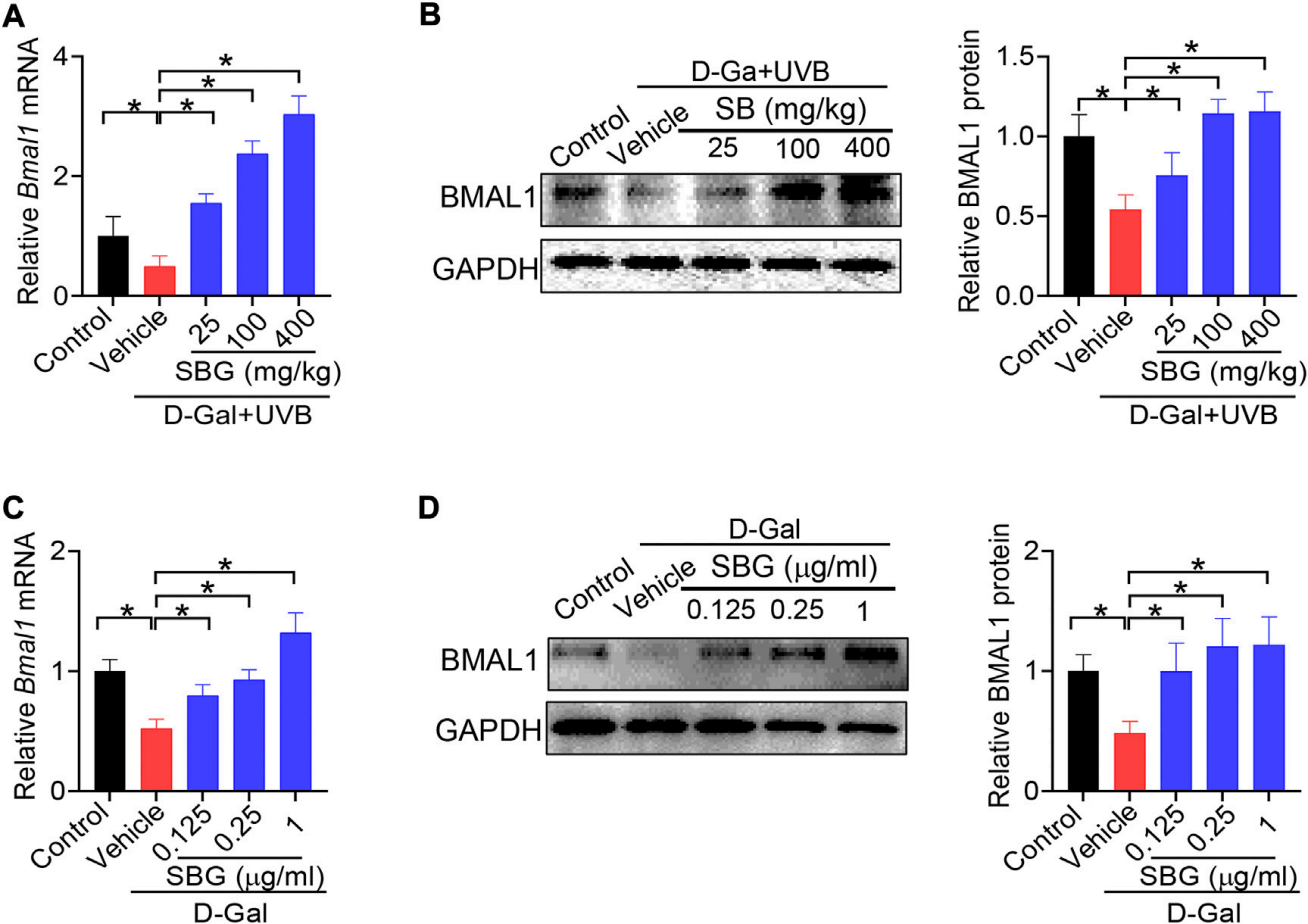
SBG up-regulates the clock gene *Bmal1* in mouse and cell models of aging**. (A)** Effects of SBG on *Bmal1* mRNA in the skin of D-gal/UVB-treated mice. **(B)** Effects of SBG on BMAL1 protein in the skin of D-gal/UVB-treated mice. **(C)** Effects of SBG on *Bmal1* mRNA in D-gal-treated NIH3T3 cells. **(D)** Effects of SBG on BMAL1 protein in D-gal-treated NIH3T3 cells. Data are mean ± SD (*n* = 7). *p < 0.05 (one-way ANOVA and Bonferroni post hoc test).

Because *Bmal1* and its upstream regulator *Rev-erbα* are involved in skin aging, we wondered whether *Bmal1* has a role in protection of skin aging by SBG. We found that SBG dose-dependently increased the BMAL1 mRNA and protein in the skin of D-gal/UVB-treated mice (Figure 8A/B). Likewise, SBG led to increases in the mRNA and protein of BMAL1 in D-gal-treated NIT3H3 cells (Figure 8C/D). Therefore, the protective effects of SBG on skin aging can be attributed to enhanced BMAL1 expression.

### SBG antagonizes REV-ERBα to induce BMAL1 expression

**FIGURE 9:**
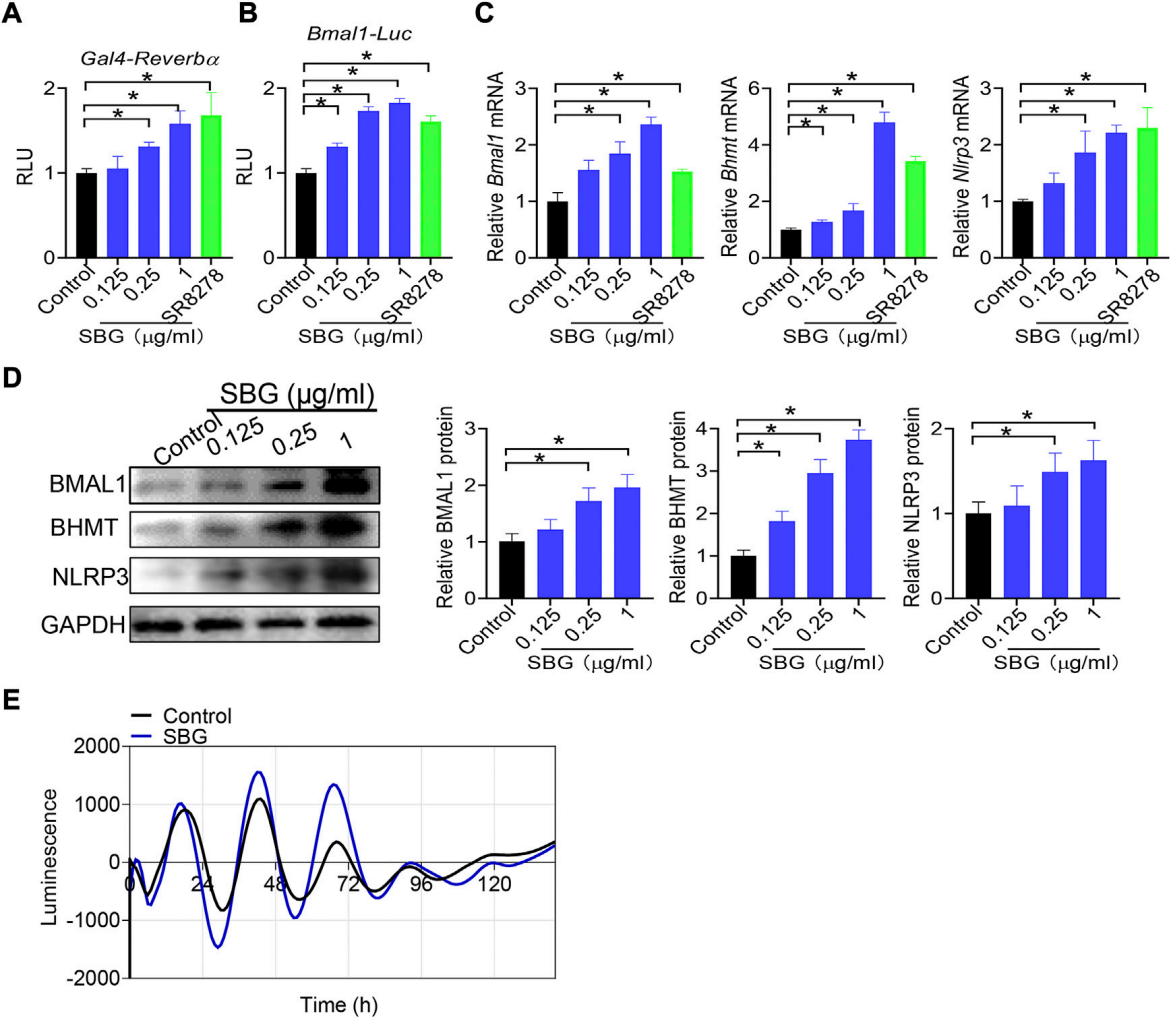
SBG antagonizes REV-ERBα to induce BMAL1 expression**. (A)** Dose-dependent effects of SBG on Gal4 luciferase activity. HEK293 cells were co-transfected with pGal4-*Rev-erbα*-LBD and pGL4.35-Luc plasmids for 24 h, followed by treatment with SBG or vehicle for 24 h. **(B)** Effects of SBG on *Bmal1-Luc* reporter activity. **(C)** Effects of SBG on the mRNA expression of *Bmal1, Bhmt and Nlrp3* in NIH3T3 cells treated with SBG or vehicle. **(D)** Effects of SBG on the protein expression of BMAL1, BHMT and NLRP3 in NIH3T3 cells treated with SBG or vehicle. **(E)** Bioluminescent recordings of *Bmal1-dLuc-*overexpressed NIH3T3 cells after SBG treatment. In panels A–B, data are mean ± SD (*n* = 6). In panels C–D, data are mean ± SD (*n* = 3). *p < 0.05 (one-way ANOVA and Bonferroni post hoc test).

*Bmal1* expression is directly regulated by REV-ERBα, a nuclear receptor whose activity can be modified by small molecules (Kojetin and Burris, 2014). We next tested whether SBG modulates *Bmal1* expression *via* REV-ERBα. We first assessed the activity of SBG in HEK293 cells expressing a chimeric receptor (i.e., the DNA-binding domain of Gal4 is fused to the LBD of *Rev-erbα*) and a Gal4-responsive luciferase reporter (Figure 9A). SBG dose-dependently inhibited the repressor activities of REV-ERBα in the Gal4 chimeric assay (Figure 9A), suggesting SBG as a REV-ERBα antagonist. Furthermore, like SR8278, SBG dose-dependently enhanced the promoter activity of *Bmal1* (−2000/+100 bp) in a luciferase reporter assay (Figure 9B). Moreover, SBG increased the mRNA and protein expression of BHMT and NLRP3 (i.e., two known targets of REV-ERBα) in addition to BMAL1 in NIH3T3 cells in a dose-dependent fashion (Figure 9C/D). In addition, SBG increased the circadian amplitude of *Bmal1* gene according to cell-based circadian assay with NIH3T3 cells expressing *Bmal1*-dLuc reporter (Figure 9E). Taken together, these findings indicated that SBG antagonized REV-ERBα to induce BMAL1 expression.

## Discussion

**FIGURE 10:**
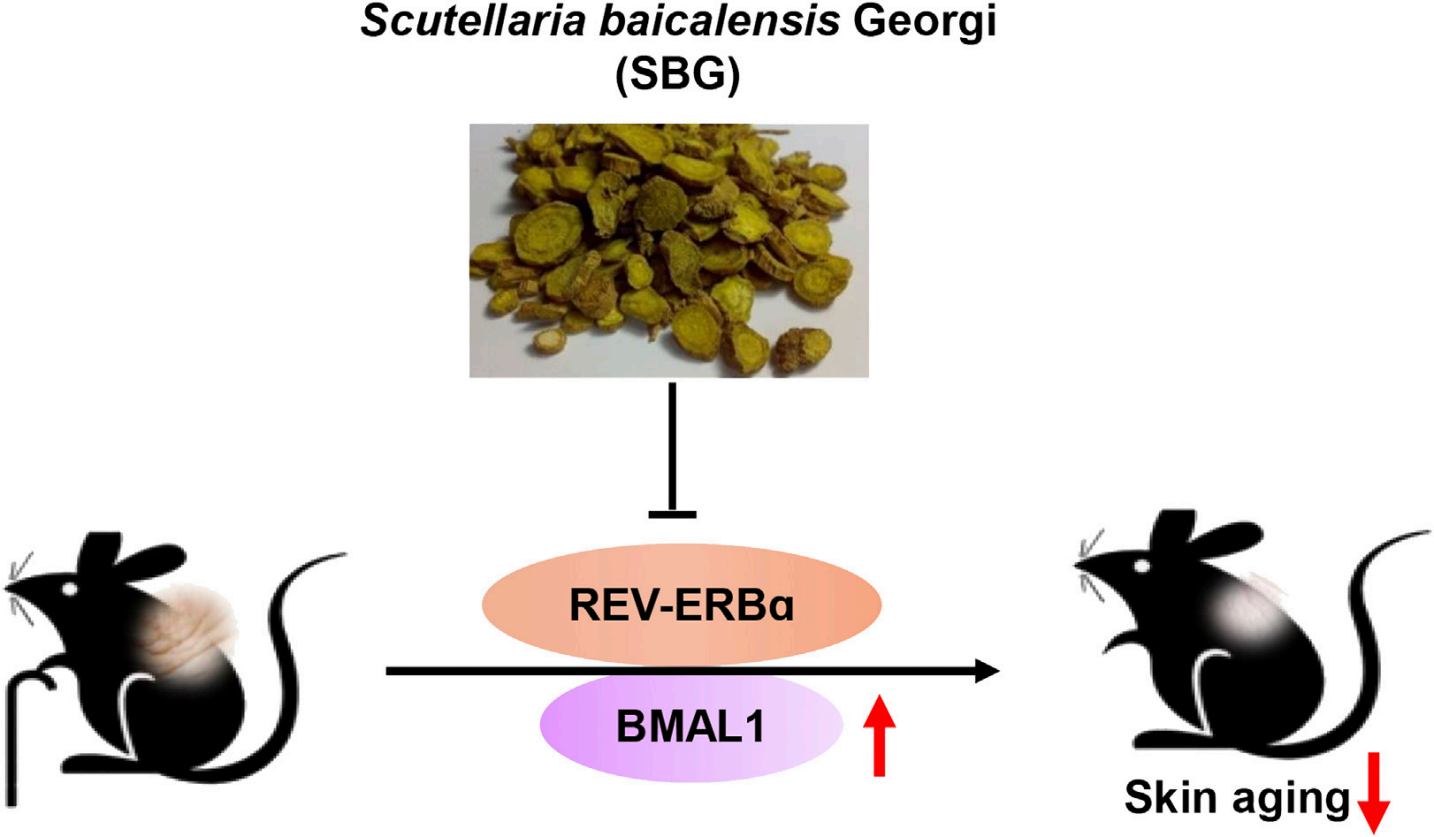
Schematic diagram showing the proposed mechanism for anti-skin aging effect of SBG.

In this study, we have identified SBG as a novel anti-skin aging agent. Compared with synthetic agents such as retinoid (Mukherjee et al., 2006), SBG is more advantageous in practical applications because it is a herbal medicine without safety concern. More importantly, we have uncovered that SBG protects against skin aging in mice by antagonizing REV-ERBα and increasing skin expression of BMAL1, an aging-inhibiting factor (Figure 10). The evidence for antagonism of REV-ERBα by SBG is strong. First, SBG dose-dependently inhibited the repressor activities of REV-ERBα in the Gal4 chimeric assay. Second, like SR8278 (a known REV-ERBα antagonist), SBG dose-dependently enhanced the promoter activity of *Bmal1* in a luciferase reporter assay. Third, SBG increased the expression of BMAL1, BHMT and NLRP3 (three known targets of REV-ERBα and repressed by REV-ERBα) in NIH3T3 cells. Fourth, SBG increased the circadian amplitude of *Bmal1* gene according to circadian assays with NIH3T3 cells expressing *Bmal1*-dLuc reporter (Figure 9). It is noteworthy that herbal extract instead of purified active compounds was used in current study. This will affect reproducibility because different sources of SBG and slight changes in extraction can lead to different composition of active compounds.

Our finding that REV-ERBα/BMAL1 (as core clock components) regulate skin aging supports the notion that skin pathophysiology is under the control of circadian clock. In fact, circadian clock has been implicated in regulation of several other types of skin diseases such as skin cancer, infections and sunburn (Duan et al., 2021). Because among clock components BMAL1 has a direct effect on skin aging, it likely acts to connect this skin disorder to circadian clock. Supporting this, *Bmal1* was markedly down-regulated in the skin of aged mice (Figure 8). We have identified REV-ERBα as a potential target for prevention and treatment of skin aging. Compared with other targets such as BMAL1, REV-ERBα is more advantageous because it is a ligand-responsive receptor whose activity can be modified by small molecules. We found that SBG is a novel REV-ERBα antagonist. However, it remains unknown which constituents in this herbal medicine are responsible for the antagonistic action. We reasoned that baicalein (a known active constituent) may have a contribution as it can induce the expression of *Bmal1,* a direct target of REV-ERBα, and shows a protective effect on skin damage (Nomura et al., 2018).

This study has unraveled that SBG promotes BMAL1 expression to protect against skin aging in mice. BMAL1 functions as an anti-aging factor by up-regulating major antioxidant enzymes such as SOD, peroxiredoxines and glutathione peroxidase (Kondratov et al., 2009; Töbelmann and Dittmar, 2021). It is noteworthy that there is a possibility that other mechanisms are also involved in the protection of skin aging by SBG considering that herbal medicines usually contain hundreds of chemical constituents. The potential mechanisms include inactivation of MAPK/AP-1 and NF-κB signaling pathways, activation of TGF-β/Smad pathway, and modulation of cyclooxygenase (COX), hypoxia-Inducible factor (HIF)-1 and inducible nitric oxide synthase (iNOS) (Domaszewska-Szostek et al., 2021; Chi and Kim, 2005; Gu et al., 2020). However, whether these mechanisms have a contribution to the SBG effect on skin aging awaits further investigations.

Mouse models of skin aging were established in current study by treatment with D-gal/UVB as previously described (Jiayi et al., 2019; Zhang et al., 2020; Cao et al., 2022). Chronic injection of D-gal, a reducing sugar, results in oxidative stress including reductions of antioxidant enzymes, inflammation and apoptosis, mimicking a natural aging process (Shen et al., 2002; Shan et al., 2009; Chen et al., 2018). UVB is the most harmful constituent of UV radiation. Chronic UVB exposure can cause excessive ROS production, abnormal elastin deposition, and impairment of collagen fibers (Starcher et al., 1999; Heck et al., 2003; Lee et al., 2021). D-gal and UVB co-treatment induces a large-scale burst of free radicals, leading to the oxidative damage in the skin and skin aging-like symptoms such as wrinkling, sagging, dryness, and erythema (Zhang et al., 2020). This animal model has been widely used to elucidate cellular and molecular changes that may have a causal role in skin aging, and to screen anti-aging drugs (Jiayi et al., 2019; Zhang et al., 2020; Cao et al., 2022).

Anti-aging effects of SBG were assessed here by measuring SOD activity, MDA/HYP levels, and MMP-1/p53 expression. SOD is a major free radical scavenger in the body, and functions to remove superoxide anions (Li et al., 2020; Park et al., 2008). MDA is a final product of oxidation, and its content can directly reflect the level of lipid peroxidation (Gil et al., 2002; Zhang et al., 2014). SOD activity and MDA level can be used as indicators of the level of organism aging which is associated with oxidative stress and ROS production (Yi et al., 2019). HYP is the most abundant amino acid in collagen, and its level indirectly reflects the total collagen content (Li and Wu, 2018; Li et al., 2016). MMP-1 is a collagenase that plays an important role in degradation of dermal collagen during skin aging (Sapna et al., 2014). Thus, HYP and MMP-1 can be used as markers of skin aging process. *P53* is a tumor suppressor gene that induces cell senescence by promoting the expression of growth suppressive genes (Bond et al., 1996; Luo et al., 2004), and is also regarded as a marker of skin aging. In fact, measurements of SOD activity, MDA/HYP levels, and MMP-1/p53 expression have been widely performed to assess aging and to screen anti-aging agents (Jiayi et al., 2019; Park et al., 2008; Hwang et al., 2012).

An important part of the skin response to stress is its ability for melatonin synthesis and subsequent metabolism (Slominski et al., 2017; Slominski et al., 2018b). Melatonin is indispensable for physiological skin functions and has a protective role against photoaging (Skobowiat, et al., 2018; Bocheva et al., 2022). However, whether SBG affects skin melatonin remains unknown. Like vitamin C, vitamin D exerts a variety of antiaging and photoprotective effects on the skin (Bocheva et al., 2021; Slominski et al., 2020). However, whether the anti-skin aging effect of SBG is comparable to that of vitamin D was unaddressed. It is noteworthy that UV radiation not only induces skin pathology, but also exert systemic effects, including activation of hypothalamic-pituitary-adrenal axis, opioidogenic effects, and immunosuppression. Thus, UV radiation has therapeutic applications in management of various diseases such as addiction, autoimmune and mood disorders (Slominski et al., 2018a).

In summary, SBG displays a pharmacological effect on skin aging based on mouse and cell models of aging. Mechanistically, SBG protects against skin aging in mice by antagonizing REV-ERBα and increasing skin expression of BMAL1, an aging-inhibiting factor.

## Abbreviation

Bmal1, brain and muscle ARNT-like one; CCGs, clock-controlled genes; Clock, circadian locomotor output cycles kaput; Crys, cryptochromes; D-gal, D-galactose; HYP, hydroxyproline; MDA, malondialdehyde; NR1D1, nuclear receptor subfamily one group D member one; Pers, periods; qPCR, quantitative polymerase chain reaction; ROS, reactive oxygen species; SA-β-gal, senescence-associated-β-galactosidase; SBG, Scutellaria baicalensis Georgi; SOD, superoxide dismutase; UV, ultraviolet

## Data Availability

The original contributions presented in the study are included in the article/Supplementary Material, further inquiries can be directed to the corresponding authors.

## Abbreviations:

Bmal1, brain and muscle ARNT–like one; CCGs, clock–controlled genes; Clock, circadian locomotor output cycles kaput; Crys, cryptochromes; D–gal, D–galactose; HYP, hydroxyproline; MDA, malondialdehyde; NR1D1, nuclear receptor subfamily one group D member one; Pers, periods; qPCR, quantitative polymerase chain reaction; ROS, reactive oxygen species; SA–β–gal, senescence–associated–β–galactosidase; SBG, Scutellaria baicalensis Georgi; SOD, superoxide dismutase; UV, ultraviolet.
