# Supplementary material for: Scutellaria baicalensis Georgi regulates REV-ERBα/BMAL1 to protect against skin aging in mice
Source: Front Pharmacol. 2022 Sep 30;13:991917. doi: 10.3389/fphar.2022.991917 (PMC9561880; doi:10.3389/fphar.2022.991917)
Supplement: Supplementary file 1 [file DataSheet1.docx]

**Supplementary material**

**Table 1:** **Main active constituents of SBG.**

|  | **Constituents** | **RT (min)** | **Transition ion pair** | **Molecular formula** | **Content (mg/g)** |
| --- | --- | --- | --- | --- | --- |
| **SBG** | Baicalin | 1.009 | 447.25→271.20 | C_21_H_18_O_11_ | 201 |
|  | Wogonoside | 1.084 | 461.2→285.20 | C_22_H_20_O_11_ | 59.7 |
|  | Baicalein | 1.616 | 271.22→123.15 | C_15_H_10_O_5_ | 4.62 |
|  | Wogonin | 2.374 | 285.20→270.20 | C_16_H_12_O_5_ | 1.62 |

**Figure S1: Representative** **chromatograms of** **wogonoside,** **baicalein, baicalin and** **wogonin (reference standards) derived from LC-MS/MS analysis.**


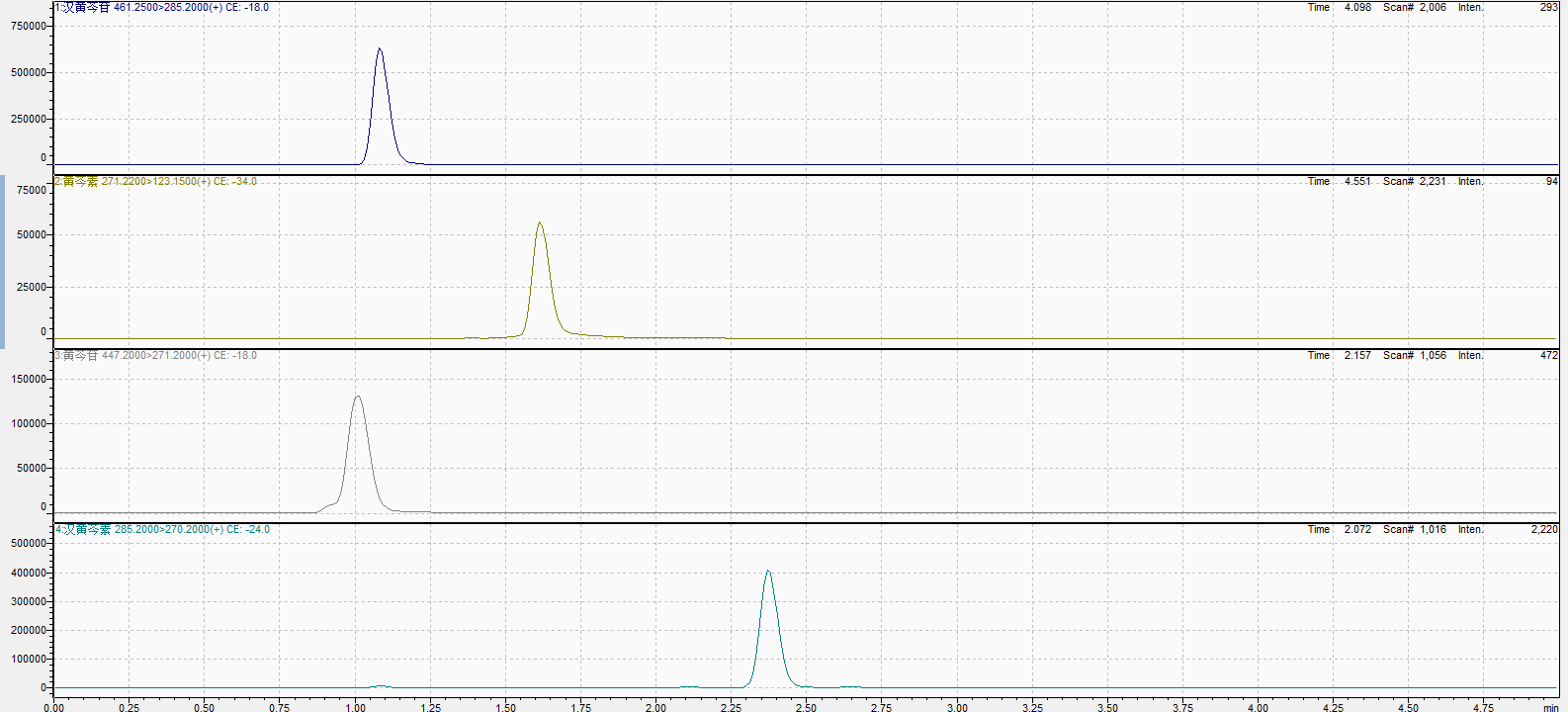


**Wogonin**

**Baicalin**

**Baicalein**

**Wogonoside**

**Figure S2: Representative chromatograms of wogonoside, baicalein, baicalin and wogonin in SBG exact derived from LC-MS/MS analysis.**


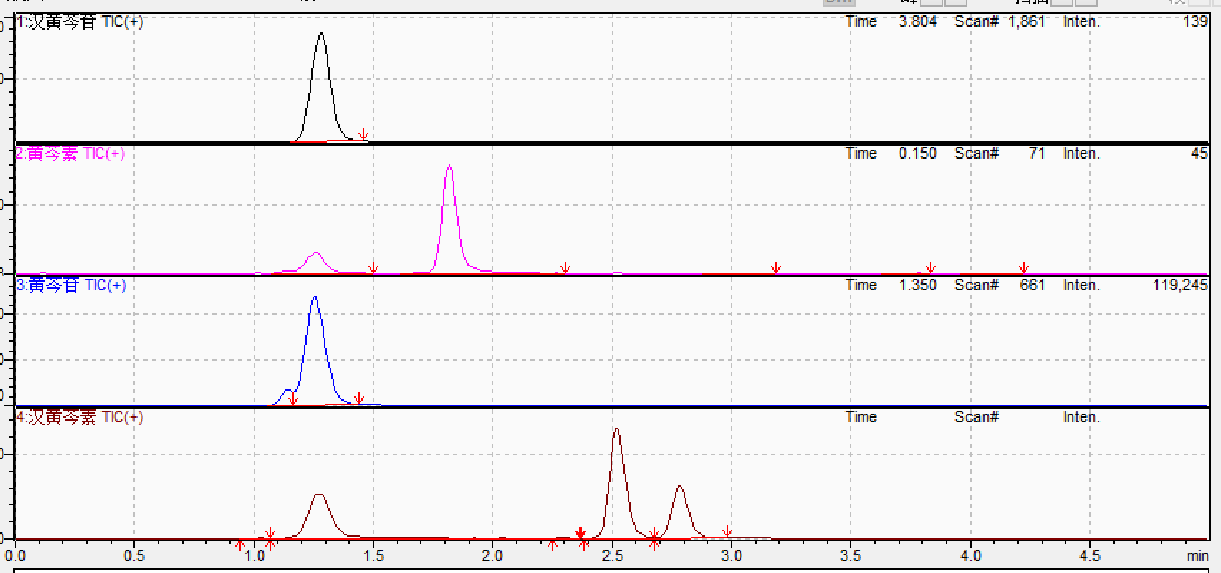


1

4

2

3

**1: Wogonoside; 2: Baicalein; 3:Baicalin; 4: Wogonin.**
